# Supplementary material for: Validating controlled attenuation parameter in the assessment of hepatic steatosis in living liver donors
Source: PLoS One. 2021 May 13;16(5):e0251487. doi: 10.1371/journal.pone.0251487 (PMC8118256; doi:10.1371/journal.pone.0251487)
Supplement: Attachment — Submitted filename: Response to reviewers. CAP study.docx [file pone.0251487.s001.docx]

Point-By- Point Response to The Comments Raised by The Reviewers:

3. In your Methods section, please provide additional information about the participant recruitment method and the demographic details of your participants. Please ensure you have provided sufficient details to replicate the analyses such as:
a) a statement as to whether your sample can be considered representative of a larger population, and
b) a description of how participants were recruited.

**3). Answer:**

**The preliminary and essential requirement for accepting potential donors to proceed with the liver transplant workup include:**

**1). Signing an informed consent for donation, (2). Being a direct relative to the patient, (3) Age between 18-50 years, (4). Body mass index (BMI) <30, (5). No previous abdominal surgery, (6). No history of alcohol or substance abuse, (7). No documented chronic medical history or on regular medication and (8). All donors were cleared by psychologist/psychiatrist from any psychiatric illness or of reduced mental capacity**

**b). Given the nature of donor process selection, the sample is not representative of the general population.**

**c). All donors were direct relatives of the patients.**

4. We note that your study involved studying potential tissue/organ transplantation donors. Please provide the following information:
a) Please state in your response letter and ethics statement whether the potential donors involved any vulnerable populations; for example, prisoners, subjects with reduced mental capacity due to illness or age, or minors.

**Answer: The following statement was added in page 6, para 2:**

“None of the potential transplant donors was from a vulnerable population (e.g, prisoners, subjects with reduced mental capacity due to illness or age, or minors) and all potential donors provided written informed consent that was freely given.”

- If a vulnerable population was used, please describe the population, justify the decision to use them as potential organ donors, and clearly describe what measures were taken in the informed consent procedure to assure protection of the vulnerable group and avoid coercion.

**Answer: NA**

- If a vulnerable population was not used, please state in your ethics statement, “None of the potential transplant donors was from a vulnerable population and all potential donors provided written informed consent that was freely given.”

**Answer: Done (Page 6, Para2)**

b) Please provide a blank example of the form used to obtain consent from donors, and an English translation if the original is in a different language.

**Answer: The English consent form has been uploaded**

c) Please indicate whether the donors were previously registered as organ donors.

**Answer: No**. **This is unfortunately not the practice in KSA**

d) Please discuss whether medical costs were covered or other cash payments were provided to the potential donor. If so, please specify the value of this support (in local currency and equivalent to U.S. dollars).

**Answer: None**

 5. Please include additional information regarding the questionnaire used for selecting living donors in the study and ensure that you have provided sufficient details that others could replicate the analyses. For instance, if you developed a questionnaire as part of this study and it is not under a copyright more restrictive than CC-BY, please include a copy, in both the original language and English, as Supporting Information.

 6.  Thank you for stating the following financial disclosure:

"The funders had no role in study design, data collection and analysis, decision to publish, or preparation of the manuscript."

At this time, please address the following queries:

1. Please clarify the sources of funding (financial or material support) for your study. List the grants or organizations that supported your study, including funding received from your institution.
2. State what role the funders took in the study. If the funders had no role in your study, please state: “The funders had no role in study design, data collection and analysis, decision to publish, or preparation of the manuscript.”
3. If any authors received a salary from any of your funders, please state which authors and which funders.
4. If you did not receive any funding for this study, please state: “The authors received no specific funding for this work.”

**Answer: No funding was provided for this study. All workup assessment measures were done as part of the transplant protocol requirements**

Please include your amended statements within your cover letter; we will change the online submission form on your behalf.

7. Thank you for submitting the above manuscript to PLOS ONE. During our internal evaluation of the manuscript, we found significant text overlap between your submission and the following previously published works, on some of which you may be an author.

https://www.sciencedirect.com/science/article/pii/S0041134518308984?via%3Dihub
https://smw.ch/article/doi/smw.2019.20077
https://bmcgastroenterol.biomedcentral.com/articles/10.1186/s12876-019-0961-9
https://journals.lww.com/eurojgh/Abstract/2017/07000/Clinical_usefulness_of_controlled_attenuation.10.aspx

We would like to make you aware that copying extracts from previous publications, especially outside the methods section, word-for-word is unacceptable. In addition, the reproduction of text from published reports has implications for the copyright that may apply to the publications.

Please revise the manuscript to rephrase the duplicated text, cite your sources, and provide details as to how the current manuscript advances on previous work. Please note that further consideration is dependent on the submission of a manuscript that addresses these concerns about the overlap in text with published work.

We will carefully review your manuscript upon resubmission, so please ensure that your revision is thorough.

**Answer: Many thanks for the valuable comment. The manuscript has been revised taking into consideration the overlaps mentioned. This has now been rectified**

5. Review Comments to the Author

Please use the space provided to explain your answers to the questions above. You may also include additional comments for the author, including concerns about dual publication, research ethics, or publication ethics. (Please upload your review as an attachment if it exceeds 20,000 characters)

Reviewer #1: Dear authors,

congrats for your paper describing a non invasive tool to investigate hepatic steatosis in the assessment of living liver donors.

I have two comments:

You're using the following scores to grade hepatic steatosis on liver biopsy - S0 (<5%), S1 (5%–33%), S2 (33%–66%), and S3 >66%. Decision making whether or not a living donor can be accepted and if yes, whether to use a right or left lobe, depends amongst other things on the liver remnant in % and the liver quality, e.g. hepatic steatosis (HS). In this regards score S1 includes a wide range of hepatic steatosis between 5 and 33 %. This range includes also a wide range of possible decisions whether or not a living donor is suitable or not at all, respectively if suitable for a right and left or only a left lobe. It seems that a subdivision of S1 would be valid, but should be at least discussed.

**Answer: We have addressed this point in the methods section (page 7, para: 2):**

Although the range in S1 is quite wide, however it remains the well-known grading system for reporting severity of steatosis. The degree of steatosis might affect the decision on donation and in most transplant centers subject will be accepted for right lobe donation if they have minimal steatosis (<5% or S0) while steatosis up to 30% (S0 or S1) can be accepted for left lobe donation. Considering subdivision of S1 however will require a separate study to validate such score.

On page 15 is written "In the 54 donors of the study by xx, ...". I guess "xx" is just a spacer for the planned citation.

**Answer: Thank you. This has been rectified (page 10, para 2):**

“In the 54 donors of the study by Yen et al, who underwent both CAP and biopsy, also showed high AUROC using a cut-off value of 257 dB/m, 100% sensitivity, and 100% NPV for CAP in evaluating HS.”

Reviewer #2: This is a very interesting paper about the potential use of the controlled attenuation parameter (CAP) in the assessment of hepatic steatosis in living liver donors.
The current gold standard is the liver biopsy but CAP may be a useful tool to avoid unnecessary biopsies, although it is still necessary due to the reported low diagnostic power of CAP in presence of severe steatosis,
This is a well-conducted prospective study, abstract, introduction, and discussion are clear and concise, aim and conclusion are coherent. Figures and tables are appropriate for the manuscript.
To-date, data on this specific topic (evaluation of steatosis in perceived healthy liver) are scarce and from relatively small case-series.
Evaluation of the necessary statistical power to use the term "validating" is required.
Other issues include:

- an evaluation of the parameters affecting the concordance between CAP and biopsy could add some value to this paper

**Answer: Many thanks for the suggestion. This has been added in the discussion (page 11, para:8):**

“Factor affecting the concordance between histology and CAP in assessment of HS like the sampling technique of the biopsy and the adequacy of the tissue are important to consider together with the presence of other liver pathology that might increase the stiffness like liver congestion and infiltration and these are less likely here as we are dealing with healthy subjects.”

- it is reported that an expert pathologist evaluates the biopsy blindly, but there is no specific indication about who performs the CAP, which is an operator-dependent test.

**Answer: One operator (Dr M. Shawkat) performed the CAP**

- why exclude left lateral hepatectomies from inclusion criteria?

**Answer: As part of our center protocol. none of the donors selected for left lateral hepatectomies needed to undergo liver biopsy as a requirement for donation.**

Minor revisions:
- please revise the language used in the Methods section of the abstract.

**Answer: Done.**

- the use of the Youden index to choose the cut-off should be mentioned first in the Method section

**Answer: Many thanks for this suggestion. This has been added to the manuscript (page:7, para: 4)**

“Youden index was used to choose the cut-off value for CAP.”

- "Another systematic review of the performance of CAP ... 1297 patients" is maybe linked to the ref 11 instead of 23.

**Answer: Many thanks for highlighting this reference mislabeling. This has now been rectified in the manuscript (page: 9, para: 2).**

- "In the 54 donors of the study by xx" should be revised

**Answer: Thank you. This has been rectified (page 10, para 2):**

**“In the 54 donors of the study by Yen et al, who underwent both CAP and biopsy, also showed high AUROC using a cut-off value of 257 dB/m, 100% sensitivity, and 100% NPV for CAP in evaluating HS.”**

- I would put the paragraph with the reference about the paper of Ayvazoglu at the beginning of the discussion since they stress the importance of the biopsy.

Answer: Many thanks for the suggestion. The authors have felt that the paragraph describing the findings of Ayvazoglu et al study is reasonably positioned in the discussion section and we prefer to keep it unchanged.
