# Supplementary material for: Validating controlled attenuation parameter in the assessment of hepatic steatosis in living liver donors
Source: PLoS One. 2021 May 13;16(5):e0251487. doi: 10.1371/journal.pone.0251487 (PMC8118256; doi:10.1371/journal.pone.0251487)
Supplement: Attachment — Submitted filename: CAP study. Response to reviewers. April 2021.docx [file pone.0251487.s002.docx]

_Dear Professor Dor,_

_Ref: PONE-D-20-31215R1
VALIDATING CONTROLED ATTENUATION PARAMETER IN THE ASSESSMENT OF HEPATIC STEATOSIS IN LIVING LIVER DONORS
PLOS ONE_

_Many thanks for the opportunity to resubmit a revised manuscript and we appreciate the valuable comments of the reviewers._

_The following are the responses to the points raised by Reviewer #2:_

1) _An evaluation of the parameters affecting the concordance between CAP and biopsy could add value to this paper._

**_Response:_** _In the earlier submission, we alluded to the factors and parameters that might impact on the concordance between CAP and biopsy. In paragraph-1 (page 11), we highlighted the fact that the donors are healthy cohorts and this might well be a factor in minimizing the potential factors that adversely impact on the concordance._

_“Factor affecting the concordance between histology and CAP in assessment of HS like the sampling technique of the biopsy and the adequacy of the tissue are important to consider together with the presence of other liver pathology that might increase the stiffness like liver congestion and infiltration and these are less likely here as we are dealing with healthy subjects.”_

2) _It is reported that an expert pathologist assessed the biopsies in a blind way. However, it is not clear who performs CAP that is an operator-dependent exam._

**_Response: The_** _Fibroscan & CAP were performed by a certified technician under the supervision of one hepatologist. The technician did more than 1000 scan on potential donors prior to his participation in this study._

3) _Why was left lateral hepatectomy chosen among the exclusion criteria?_

**_Response:_** _As per our department transplant protocol, liver biopsy is not a requirement from donors of left lateral segment. The liver biopsies were only for right lobe donors._

4) _Please revise the language used in the methods section of the abstract._

_Response: Many thanks for the suggestion. These parts have been proofread._

5) _The use of the Youden index to identify the cut-off should be mentioned with priority in the Methods section._

**_Response:_** _This point is now highlighted in the last paragraph (page 7)._

6) _"Another systematic review of the performace of CAP ... 1297 patients" is possibly linked to ref 11 instead of 23 ?._

**_Response:_** _Many thanks for the suggestions. This has now been corrected (para: 3, page 10):_

_Another systematic review, involving 1297 patients, evaluated the performance of CAP in the diagnosis and staging of HS and biopsy-proven NAFLD._^(11)^

7) _"In the 54 donors of the study by xx" should be revised._

**_Response:_** _Thank you. This has now been revised (para 4: page 10):_

_“ A study of 54 donors by Yen et al, who underwent both CAP and biopsy, also showed high AUROC using a cut-off value of 257 dB/m, 100% sensitivity, and 100% NPV for CAP in evaluating HS”._

8) _The paragraph with the reference regarding the paper of Ayvazoglu should find a place earlier in the Discussion given that they clarify the importance of the biopsy._

**_Response:_** _Many thanks for the suggestion. This paragraph has now moved up to page 9 (para: 2). The order of the references has now changed upon the transfer of this paragraph_

_We hope that the points raised by the reviewers have been addressed and once again, many thanks for the opportunity to resubmit the manuscript._

_Best wishes_

_Khalid Bzeizi_

_-_
